# Immune infiltration analysis reveals immune cell signatures in salivary gland tissue of primary Sjögren’s syndrome

**DOI:** 10.3389/fmed.2023.1033232

**Published:** 2023-01-18

**Authors:** Hongxiao Gong, Xiaoting Qiu, Pingping Li, Runzhi Zhao, Beijia Wang, Ling Zhu, Xingxing Huo

**Affiliations:** ^1^Department of Scientific Research, Experimental Center of Clinical Research, The First Affiliated Hospital of Anhui University of Chinese Medicine, Hefei, Anhui, China; ^2^Department of Otolaryngology, The First Affiliated Hospital of Anhui University of Chinese Medicine, Hefei, Anhui, China

**Keywords:** primary Sjögren’s syndrome, immune infiltration, salivary glands, RNA-seq sequencing, cross-species analysis

## Abstract

**Introduction:**

Mouse models are the basis for primary Sjögren’s syndrome (pSS) research. However, the depth of comparisons between mice and humans in salivary gland (SG) immune cells remains limited.

**Methods:**

The gene expression profiles of SGs from normal subjects and pSS patients were downloaded from the Gene Expression Comprehensive Database. The proportion of infiltrating immune cell subsets was then assessed by cell type identification by estimating relative subsets of RNA transcripts (CIBERSORT). An experimental Sjögren’s syndrome (ESS) mouse model was successfully constructed using SG protein. Based on mouse SG tissue RNA-Seq data, the seq-ImmuCC model was used to quantitatively analyze the compositional ratios of 10 immune cells in pSS patients and mouse model SG tissues.

**Results:**

Computed and obtained 31 human data samples using the CIBERSORT deconvolution method. The immune cell infiltration results showed that, compared to normal human SG tissue, the content of gamma delta T cells was significantly different from naive CD4^+^ T cells and significantly increased, while the plasma cell content decreased. Principal component analysis indicated differences in immune cell infiltration between pSS patients and normal subjects. Meanwhile, for ESS model mouse data analysis, we found that the proportion of macrophages increased, while the proportion of CD4^+^ T cells, B cells, and monocytes decreased. Furthermore, we found that the proportion of monocytes was decreased, while the proportion of macrophages was increased in the SG tissues of pSS patients and model mice. The infiltration of CD4^+^ T, CD8^+^ T, and B cells also showed some differences.

**Discussion:**

We comprehensively analyzed SG immune infiltration in pSS patients and model mice. We demonstrated conserved and nonconserved aspects of the immune system in mice and humans at the level of immune cells to help explain the primary regulation of immune mechanisms during the development of Sjögren’s syndrome.

## Introduction

Primary Sjögren’s syndrome (pSS) is a common systemic autoimmune disease. Statistics have shown that its incidence in women is much higher than in men, with a 9–14:1 ratio (1). This disease has clear characteristics, manifested as the loss of secretory function caused by the infiltration of lymphocytes in the exocrine glands. Among them, lacrimal and salivary gland dysfunctions are the most common (2). The pathogenesis of the disease is not entirely clear, which may be related to immune damage, environment and genetics (3). The abnormal number of immune cells in patients’ target organs and blood is also essential in pSS occurrence and aggravation (4).

The cell type identification by estimating relative subsets of RNA transcripts (CIBERSORT) algorithm can assess immune cell infiltration in tissues based on gene expression datasets (5). Many studies have used this algorithm to explore the composition of immune cells in disease (6). RNA sequencing (RNA-seq) enables analysis of the entire transcriptome to reveal changes in entire signaling networks and potentially predict novel genes of significance. Extracting tissue immune environment from transcriptome data has gradually become a research hotspot, but it rarely involves mouse RNA-seq data. The seq-ImmuCC model was recently developed to describe the composition of major immune cells in different tissues or organs from mouse RNA-Seq data (7).

Mouse models are invaluable tools for characterizing signaling pathways and biomedical research, and most studies on pSS are based on mice models. However, immunological differences exist between humans and mice (8, 9). Through literature review and previous research of the research group, C57BL/6 mice are found to be a typical animal model of antigen induced SS (10–12). We performed immune infiltration analyses of the pSS human salivary gland (SG) CIBERSORT (13) and pSS C57BL/6 mouse seq-ImmuCC models. Then, we performed a comprehensive comparison to determine the cellular characteristics of humans and mice and the evolutionary similarities of their immune systems. Our current results might provide insights into the immunomodulatory mechanism of pSS.

## Materials and methods

### Data sources

The gene expression profile chip dataset of SGs from normal subjects and pSS patients (GSE40611) was retrieved from the gene expression omnibus (GEO) database.^1^ Thirty-one samples were available, including 16 normal subjects and 15 pSS patients’ SG tissues.

### Evaluation of immune cell infiltration patterns in SG tissue of pSS patients

We used Perl to organize the gene expression data into a gene matrix with rows with genes and columns with samples. The mRNA expression profile matrix of SG tissue of normal subjects and pSS patients was corrected by the “BioManager” and “limma” R packages. The deconvolution analysis was performed on the expression matrix of human immune cell subtypes with CIBERSORT. The relative proportions of 22 immune cells in the tissue were calculated, and a *p*-value was obtained for each sample. Samples were screened according to *p* < 0.05 and the immune cell composition matrix of salivary gland tissue from 16 normal subjects and 15 pSS patients. The R language and related packages were used to draw histograms and immune cell expression heatmaps of each immune cell composition ratio in the two groups. The “corrplot” R package was used to analyze the correlations of immune cells in the SGs of pSS patients and draw heatmaps; the “vioplot” R package was used to compare and analyze the proportion of SG immune cells in normal subjects and pSS patients and draw a violin diagram.

### Construction of Sjögren’s syndrome mouse model

Specific pathogen-free 6-week-old female C57BL/6 mice were purchased from VitalLiver Laboratory Animal Technology. Mice were housed at constant temperature and humidity and fed *ad libitum*. All animal studies were performed following protocols approved by the Animal Experiment Ethics Review Committee of the Anhui University of Traditional Chinese Medicine (Animal Ethics Number: AHUCM-mouse-2021082). To construct the SG protein-induced pSS (ESS) mouse model (14), the SG protein emulsion was subcutaneously injected into the dorsal and caudal bases of the mice to establish ESS. We regularly detected the salivary flow of mice at 0, 6, and 8 weeks (15), combined with HE staining histopathological score to determine the structure of the mouse model (16). Mice that were unsuccessfully modeled were eliminated, and the successfully established pSS mice model was characterized by clinical symptoms and significantly decreased salivary flow. For RNA-seq, mice were randomly divided into two groups: control (*n* = 3) and ESS (*n* = 3) groups.

### RNA-seq

The SGs of three control and six model mice were selected for RNA-seq. Mice in the model group showed significantly reduced salivary secretion and evident inflammation foci in the pathological section specimens (RNA-zzseq method in Supplementary material).

### Statistical methods

The R (version 4.0.2) software and ‘‘Bioconductor^2^ ‘’ package were used for statistical analysis. For data sorting, the list deletion method was used for processing. The entire column was deleted for samples lacking any single value, which is unsuitable for statistical analysis. The ‘‘corrplot’’ R package was used to detect and draw the correlations between different immune cells in the SG tissue of pSS patients; the ‘‘vioplot’’ R package was used to calculate and draw the proportion of different immune cells in the two groups. We used the newly developed seq-ImmuCC^3^ to process the off-camera data, as previously described (7, 17). Pearson’s correlation coefficients were used to measure the degree of linear fit and determine the agreement between the relative scores of immune cell types. The root mean square error (RMSE) was used to assess estimation bias. Heatmaps were constructed using the “pheatmap” R package. All statistical analyses were performed using R software (18). Salivary secretion level was analyzed with an Unpaired two-tailed *t*-test. Normality and homogeneity of variance were verified using the Shapiro–Wilk test and Levene’s test, respectively. Data are described by means ± SEMs. For all statistical tests, *P* < 0.05 was considered statistically significant.

## Results

### Immune cell infiltration in pSS patients and normal subjects

**FIGURE 1:**
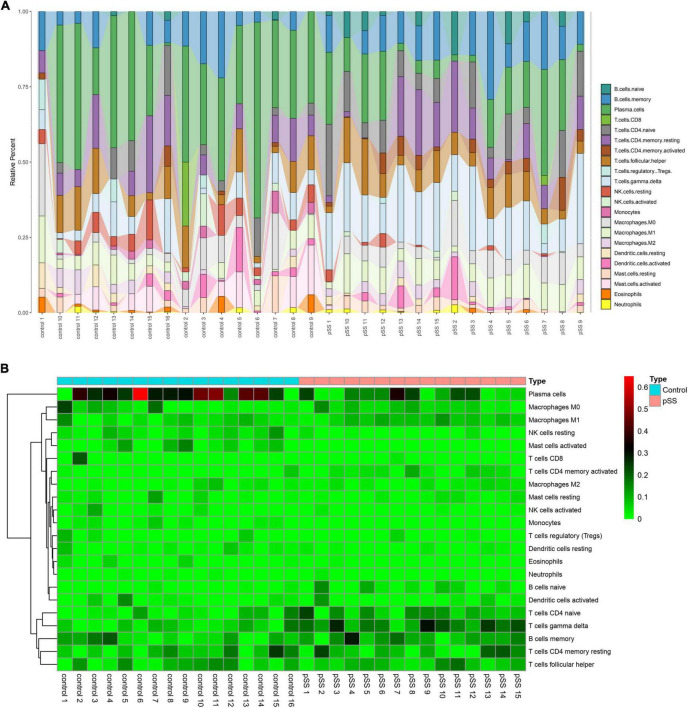
Salivary gland immune cell infiltration in pSS patients and normal individuals. **(A)** Histograms (corresponding color modules indicate the type, and size indicates the proportion of the total immune cells) and **(B)** heatmap of the proportions of 22 immune cells in the salivary glands of pSS patients and normal individuals.

The CIBERSORT deconvolution method was used to calculate and obtain 31 credible samples, including 16 normal and 15 pSS samples. The immune cell infiltration results showed that, compared to the normal group, CD4 naive T, gamma delta T, memory B, and memory CD4 T cells significantly increased in the pSS group, T-cells CD8 did not clearly change, while plasma cells significantly decreased. Dendritic cells resting and dendritic cells activated did not clearly increased (Figure 1A). The infiltration ratio of 22 immune cell types is presented in Figure 1B.

### Human immune cell correlation analysis

**FIGURE 2:**
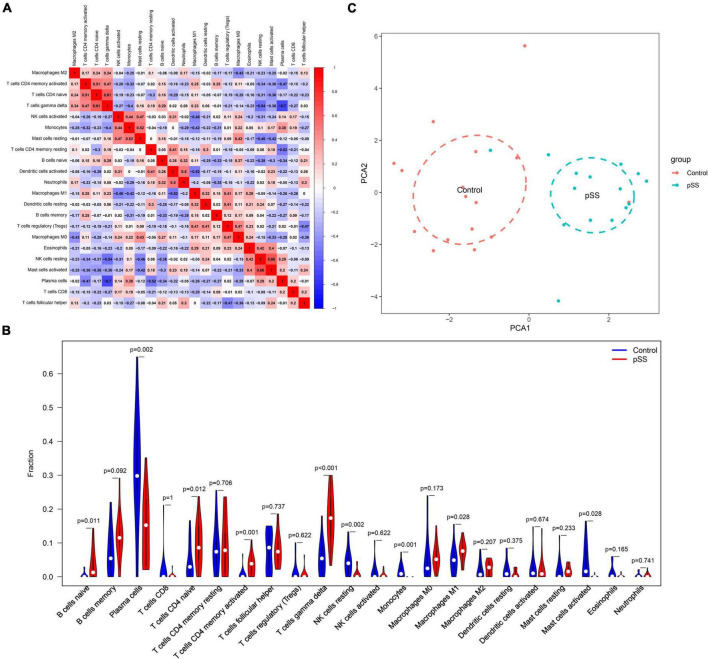
Correlation of salivary gland immune cells between pSS patients and normal subjects. **(A)** Correlation between immune cell subsets. There was a significant negative correlation between gamma delta T and plasma cells. **(B)** Comparison of 22 immune cell subsets in the salivary glands of pSS patients and normal subjects. The proportion of gamma delta T-cells and M1 macrophage infiltration was higher in pSS patients (*p* < 0.05 was considered statistically significant). **(C)** Principal component analysis plot of salivary gland immune cell infiltration in pSS patients and normal individuals. Blue is the normal group; red is the pSS group.

The immune cell correlation analysis showed a significant positive correlation between gamma delta T-cells and CD4 naive T-cells (*r* = 0.61, *p* < 0.05), and resting NK cells and activated Mast cells (*r* = 0.66, *p* < 0.05). A significant negative correlation was detected between gamma delta T and plasma cells (*r* = −0.7, *p* < 0.05) (Figure 2A). The violin diagram showed that pSS patients have abundant macrophage infiltration compared to normal subjects. Additionally, compared to normal subjects, pSS patients have significantly increased content of naive B, activated memory CD4 T, and gamma delta T-cells. On the other hand, the content of plasma cells, resting NK cells, and monocytes significantly decreased (Figure 2B). Finally, the principal component analysis (PCA) based on infiltrating immune cell types revealed discriminative results between pSS and healthy tissues (Figure 2C).

### Establishment of the ESS model

**FIGURE 3:**
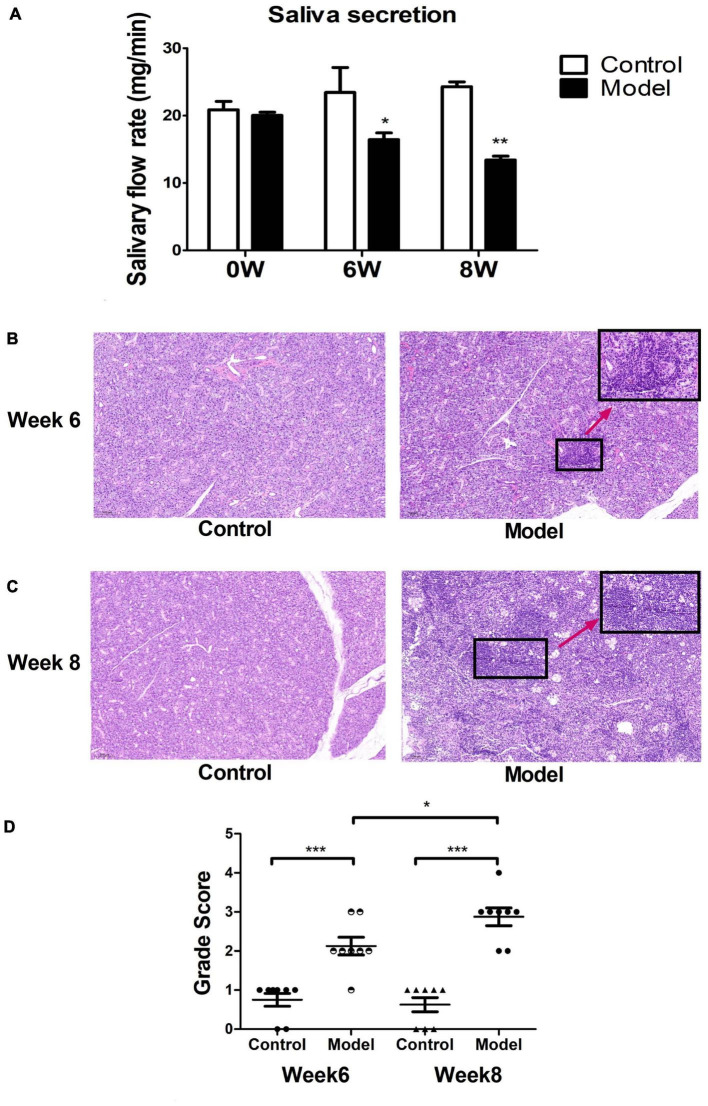
Mouse modeling results. **(A)** The saliva secretion of ESS model mice was significantly lower than the normal group. Measured at weeks 6 and 8 ($\bar{\chi }$ ± s, *n* = 3), ***p < 0.001, **p < 0.01, and *p < 0.05 vs. control. Histological observation of salivary gland injury at week 6 **(B)** and **(C)** 8 in ESS mice and controls. **(D)** Histological scoring of salivary gland lesions at week 6 and week 8 in ESS mice and controls ($\bar{\chi }$ ± s, *n* = 8), ***p < 0.001, **p < 0.01, and *p < 0.05 vs. controls.

Most C57BL/6 mice in the model group developed pSS clinical symptoms after immunization with allogeneic SG protein. We found that the saliva secretion of model mice significantly decreased from week six after immunization and the overall salivary secretion leveled off, different from unimmunized controls (Figure 3A). After histological analysis of SG tissue sections of the ESS model and control mice, we found that the SGs of ESS mice had more lymphocyte infiltration after 6 weeks of immunization (Figure 3B). Moreover, multiple lymphocyte foci were detected after 8 weeks of immunization. We also observed that the acinus of model mice was damaged, different from the control group (Figure 3C). Then, we used a scoring system and found that the infiltration severity was quantified to achieve an intuitive expression effect. The histological scores in the pSS model group were significantly higher than those in the control group. The scores in the model group tended to increase over time (Figure 3D). Altogether, these results demonstrated that the model was successfully established.

### Immune cell infiltration in mice

**FIGURE 4:**
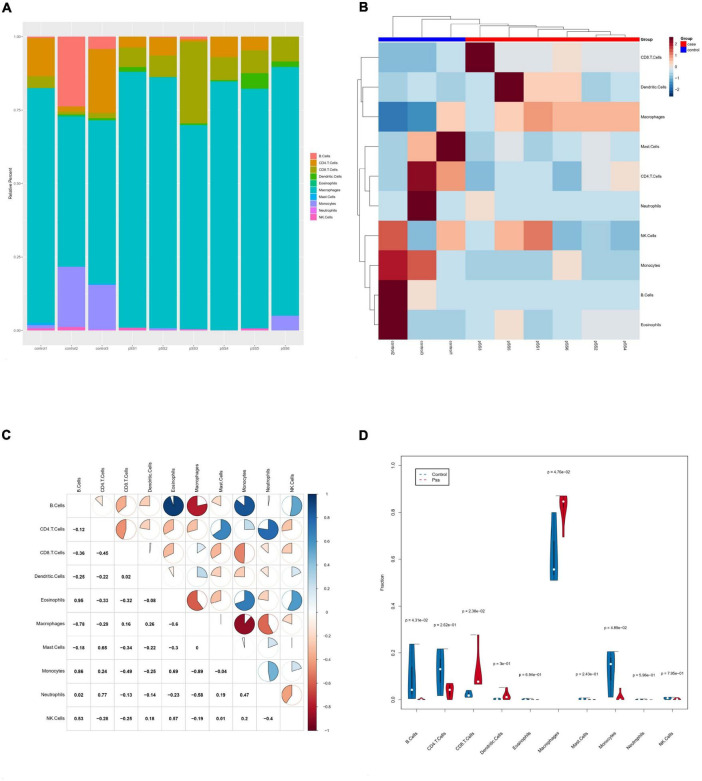
Infiltration of salivary gland immune cells in ESS mice. **(A)** The composition of 10 immune cells in salivary gland tissue based on ImmuCC assay. **(B)** Clustering heatmap of salivary gland immune cells in ESS model mice and control mice. **(C)** Correlations between immune cell subsets. There was a negative correlation between monocytes and macrophages (*p* < 0.05 was considered statistically significant). **(D)** Comparison of infiltrating immune cell subsets in salivary gland tissues of ESS model and control mice. The proportion of macrophage infiltration was higher in ESS model mice. Macrophages increased in salivary gland tissue of ESS lesions. Blue is the normal group; red is the ESS group.

Based on mouse salivary gland tissue RNA-Seq data and the seq-ImmuCC model, ten types of immune cells were analyzed in mice’s SG tissue: B cells, CD4^+^ T cells, monocytes, macrophages, neutrophils, CD8^+^ T cells, NK cells, mast cells, dendritic cells, and eosinophils. We detected abundant macrophage infiltration in all SG tissues (Figure 4A). The cluster analysis showed that the immune cell infiltration of the ESS model and control mice had evident inter-group differences (Figure 4B). The immune cell correlation analysis showed a significant positive correlation for eosinophils and B cells (*r* = 0.95, *p* < 0.05), monocytes and B cells (*r* = 0.86, *p* < 0.05), neutrophils and CD4^+^ T cells (*r* = 0.77, *p* < 0.05), monocytes and eosinophils (*r* = 0.69, *p* < 0.05). Meanwhile, macrophages and B cells (*r* = −0.78, *p* < 0.05), macrophages and eosinophils (*r* = −0.6, *p* < 0.05), monocytes and macrophages (*r* = −0.89, *p* < 0.05) had a significant negative correlation (Figure 4C). The violin diagram showed that, compared to control mice, the SGs of ESS model mice presented significantly increased proportions of macrophages and CD8^+^ T cells, while CD4^+^ T cells, monocytes, and B cells significantly decreased (Figure 4D).

### Comprehensive analysis of immune cell infiltration in pSS patients and ESS model mice

The immune infiltration analysis of pSS patients and ESS model mice showed similarities and differences in the composition of immune cells. For human data, we found that gamma delta T-cells, CD4 naive T-cells, naive B cells, M1 macrophages, and activated memory CD4^+^ T cells significantly increased, while plasma cells, resting NK cells, and monocytes significantly decreased (Figure 2B). In model mice, the number of macrophages and CD8^+^ T cells significantly increased, while the proportion of CD4^+^ T cells, B cells, and monocytes decreased (Figure 4D). Combining these results, we found that the proportion of monocytes was reduced in pSS patients (*p* = 0.001) and SG tissue of model mice (*p* = 0.0489). Additionally, macrophages (*p* = 0.0476) significantly increased in model mice, M1 macrophages (*p* = 0.028) significantly increased in pSS patients, while M0 (*p* = 0.173) and M2 macrophages (*p* = 0.207) did not clearly increased. The proportion of CD8^+^ T cells (*p* = 0.0238) only increased in model mice. On the other hand, the contents of CD4^+^ T cells (*p* = 0.0262) and B cells (*p* = 0.0431) decreased in the tissue of model mice by varying degrees but presented an upward trend in pSS patients.

## Discussion

pSS is a common autoimmune disease in middle-aged and older women that targets exocrine glands, such as SGs, and might involve multiple organs and systems. Many studies have shown that the proportion of immune cells in pSS patients differs from normal subjects, which is also an important factor in its occurrence and aggravation. High-throughput analytical techniques such as transcriptomics and expression microarray analysis allow the gain of insight into immune cell composition changes. A unique feature of this study was the correlation of human gene expression data with ESS C57BL/6 mice. The comprehensive analysis of the data enabled the characterization of cells in human and mouse models and might provide some insights into the mechanisms of pSS immune regulation.

In the human analysis, expression matrices of human immune cell subtypes were deconvoluted and plotted using R. In the mouse analysis, we first constructed an ESS mouse model. Then, we identified changes in saliva volume and lymphocyte infiltration in SG tissue to determine the model’s success. Next, we collected mouse SG tissues, sequenced their RNA (RNA-seq), processed the results by seq-ImmuCC to conduct a comprehensive analysis. By summarizing the cellular characteristics between humans and mice, we expanded the understanding of pSS-related regulatory mechanisms.

The immune infiltration analysis showed that mouse and human immune cells have certain common features. The proportion of monocytes in submandibular gland tissues of pSS patients and ESS mice decreased. In addition, macrophages showed the same change trend. However, the proportion of CD8^+^ T cells only increased in ESS mice. The ratio of CD4^+^ T cells to B cells decreased in the ESS mouse model but increased in humans. For human data, we found that gamma delta and naive CD4 T cells were positively correlated with plasma cells. Besides, naive B cells, activated memory CD4, and gamma delta T-cells were significantly increased, while plasma cells, resting NK cells, and monocytes significantly decreased. Furthermore, the PCA results suggested that immune cell infiltration can be used to differentiate pSS patients from normal individuals. For mouse data analysis, we found that macrophages were negatively correlated with B cells, eosinophils, and monocytes, and the number of macrophages increased. The trend of CD4^+^ T cells, monocytes, and B cells significantly decreased.

B lymphocytes are generated by differentiation of hematopoietic stem cells, mature in the bone marrow, and finally activate in secondary lymphoid organs. It has been suggested that the increased frequency of autoreactive antibody expression by mature naive B cells and impaired peripheral B cell tolerance in pSS patients might be involved in the pSS pathogenesis (19, 20). Plasma cells are terminally differentiated B cells that perform multiple functions by producing antibodies and other cytokines. We observed that the number of naive B cells in patients with pSS increased, while the number of plasma cells decreased. It may be related to the early damage of B cells in patients with pSS. Macrophages are innate immune cells and play many essential roles, including host defense, tissue homeostasis, and regulation of inflammatory responses (21, 22). Macrophages are also an important part of inflammatory cells infiltrating salivary glands. Many studies have shown that macrophages are involved in the pathological damage of salivary glands (23–25). The macrophages in the salivary glands are pathogenic and can secrete related cytokines. Additionally, patients with significant macrophage infiltration in SGs have increased swelling (23), consistent with the increased numbers of macrophages in our current results. Meanwhile, they are known to be involved in pSS pathogenesis. Increasing studies have suggested that infiltrating monocytes and macrophages are phenotypically altered in autoimmune disease and have been implicated in both mice and humans (26–29). The monocytes in pSS patients are inefficient in the clearance of apoptotic cells, which is also associated with the production of pro-inflammatory cytokines such as IL-6, TNF-α, IL-10, transforming growth factor-β, and interferon-α (30–32).

Furthermore, CD8^+^ T lymphocytes are a complex group of lymphocytes with different phenotypes. Studies have shown that activated CD8^+^ T lymphocytes aggregate around apoptotic acinar epithelial cells, and CD8^+^ T cells infiltrating glands show cytotoxicity unaffected by CD4^+^ T cells (33). As the disease progresses, the proportion of CD8^+^ T cells in pSS patients is significantly increased compared to normal subjects (34), consistent with our results. Memory CD4^+^ T cells are highly associated with autoimmune diseases due to their long-lived properties, efficient responses to antigens and potential to mediate recurrent autoimmune responses (35). However, many key questions about the potential contribution of memory CD4^+^ T cells to autoimmune disease remain unanswered. Some studies have shown that the relative proportion of memory CD4^+^ T increases in pSS patients compared to normal controls (36), consistent with our human immune infiltration results. However, the specificity of memory T-cells and pSS is different, and their relationship is still not fully understood. Moreover, some scholars have found that the reduction of lymphocytes in pSS patients is related to the premature senescence of naive CD4^+^ T cells in the body, which might also explain the reduction of CD4^+^ T lymphocytes in the mouse data (37). Determining the immunobiological roles of CD4^+^ T and CD8^+^ T cells in pSS is critical for developing targeted therapies based on these cells. Gamma delta (γ/δ^+^) T-cells account for about 2–5% of peripheral blood T lymphocytes and are mainly distributed in mucosa-associated lymphoid tissues. Their immune functions are innate and adaptive immunity, participating in tissue homeostasis and infection monitoring (38). It has been reported that the proportion of activated cells in γ/δ^+^ T-cell subsets in peripheral blood of pSS patients is significantly higher than that of controls, and the frequency of activated cells is related to disease course (39), consistent with our results. These results also suggested that this T-cell subset may play a role in the pathological immune response encountered in pSS.

However, our study also has some limitations. First, the seq-ImmuCC model only covers ten cell types. For example, gamma delta T-cells, which play an important role in the immune system, are not included, and further improvement is required. Additionally, the human experiment was based on bioinformatic immune infiltration analysis of transcriptomic signatures from public datasets, and further studies are needed to confirm the findings observed in human biopsies.

## Conclusion

Herein, we performed immune infiltration analysis in pSS humans and model mice and predicted abnormal disease-specific immune infiltration patterns to reveal the underlying immune pathogenesis of pSS. We found that monocyte levels were reduced in pSS patients and model mice. However, further experiments are required to demonstrate that these cells perform tissue damage in pSS, as our current results suggest. Therefore, we provided insights for disease-modifying immunotherapies by targeting specific immune cells.

## Data availability statement

DAS Publicly available datasets were analyzed in this study. This data can be found here: GEO database, GSE40611. The original contributions presented in this study are publicly available. This data can be found here: NCBI SRA database, under accession PRJNA839202.

## Ethics statement

The animal study was reviewed and approved by the Ethics Review Committee for Animal Experimentation, Anhui University of Chinese Medicine.

## Author contributions

XH conceived and designed the experiments, performed the experiments, approved the final version, and analyzed the data. XQ and HG contributed to the drafting of the submitted article, the accuracy of the data analysis, and analysis and interpretation of the data. PL, RZ, and BW contributed to the acquisition of reagents, materials, and analysis tools. XH and LZ contributed to perform the validation experiment and revised the manuscript. All authors contributed to the article and approved the submitted version.

## Footnotes

1
https://www.ncbi.nlm.nih.gov/

2
https://www.bioconductor.org/

3
http://218.4.234.74:3200/immune/
