# Supplementary material for: Immune infiltration analysis reveals immune cell signatures in salivary gland tissue of primary Sjögren’s syndrome
Source: Front Med (Lausanne). 2023 Jan 18;10:1033232. doi: 10.3389/fmed.2023.1033232 (PMC9889644; doi:10.3389/fmed.2023.1033232)
Supplement: Supplementary file 1 [file Table_1.docx]

**RNA-seq**

RNA from total samples was isolated and purified using TRIzol (Invitrogen, CA, USA) following the manufacturer’s instructions. The quantity and purity of total RNA were checked with NanoDrop ND-1000 (NanoDrop, Wilmington, DE, USA). The RNA integrity was detected by Bioanalyzer 2100 (Agilent, CA, USA) and verified by agarose electrophoresis. A concentration > 50 ng/μL, RIN value > 7.0, OD_260/280_ > 1.8, total RNA > 1μg was used as threshold for downstream experiments. PolyA (polyA)-bearing mRNA was specifically captured by two rounds of purification using oligo(dT) magnetic beads (Dynabeads Oligo (dT), Cat. No. 25-61005, Thermo Fisher, USA). The captured mRNA was fragmented using a Magnesium Fragmentation Kit (NEBNext® Magnesium RNA Fragmentation Module, Cat. No. E6150S, USA) at 94 °C for 5-7 min. The cDNA was synthesized from the fragmented RNA using reverse transcriptase (Invitrogen SuperScript™ II Reverse Transcriptase, Cat. No. 1896649, CA, USA). Then, E. coli DNA polymerase I (NEB, Cat. No. m0209, USA) and RNase H (NEB, Cat. No. m0297, USA) were applied for double-strand synthesis. These complex DNA and RNA duplexes were converted into DNA double-strands. The dUTP Solution (Thermo Fisher, Cat. No. R0133, CA, USA) was incorporated into the second strand to blunt the ends of the double-stranded DNA. Then, the “A” base was added to each end to connect to a “T” base linker at the end; the fragment size was screened and purified by magnetic beads. The second strand was digested with UDG enzyme (NEB, cat. no. m0280, MA, US), and the PCR was conducted to form a library with a fragment size of 300 ± 50bp: pre-denaturation at 95 °C for 3 min; denaturation at 98 °C for 8 cycles of 15 s each; annealing at 60 °C for 15 s; extension at 72 °C for 30 s; final extension at 72 °C for 5 min. Finally, we performed the paired-end sequencing using Illumina Novaseq™ 6000 (LC Bio Technology CO., Ltd. Hangzhou, China) in PE150 sequencing mode according to standard procedures.
